# Macrophage migration inhibitory factor promotes vasculogenic mimicry formation induced by hypoxia via CXCR4/AKT/EMT pathway in human glioblastoma cells

**DOI:** 10.18632/oncotarget.18673

**Published:** 2017-06-27

**Authors:** Xing Guo, Shugang Xu, Xiao Gao, Jian Wang, Hao Xue, Zihang Chen, Jinsen Zhang, Xiaofan Guo, Mingyu Qian, Wei Qiu, Gang Li

**Affiliations:** ^1^ Department of Neurosurgery, Qilu Hospital of Shandong University, Jinan, Shandong Province, P.R. China; ^2^ Brian Science Research Institute, Shandong University, Jinan, Shandong Province, P.R. China; ^3^ Department of Biomedicine, University of Bergen, 5009-Bergen, Norway; ^4^ Department of Neurosurgery, Dezhou People's Hospital, Dezhou, Shandong Province, P.R. China

**Keywords:** hypoxia, vasculogenic mimicry, MIF, CXCR4, glioblastoma

## Abstract

Macrophage migration inhibitory factor (MIF) is over-expressed and secreted in various cancer cells in particular in response to hypoxia. Recent studies have shown that, under hypoxic conditions, glioblastoma (GBM) cells display the ability to drive blood-perfused vasculogenic mimicry (VM). The aim of this study was to investigate the underlying mechanism of MIF in the regulation of hypoxia-induced VM in GBM cells. By analyzing clinical specimens, we observed the co-localization of MIF, C-X-C motif chemokine receptor 4 (CXCR4) and VM in hypoxic regions of gliomas. *In vitro*, the exposure of GBM cells (U87 and U251) to hypoxia increased the expression of MIF and CXCR4 and induced VMs. Other data demonstrated that ectogenic rhMIF promoted VMs in GBM cells and knock-down endogenous MIF attenuated hypoxia-induced VMs. In addition, we demonstrated that MIF augmented VM formation ability by enhancing the epithelial mesenchymal transition (EMT) through the CXCR4-AKT pathway. Moreover, *in vivo*, the subcutaneous injection of rhMIF resulted in the progression of EMT and VMs formation. On the contrary, CXCR4-AKT pathway inhibitors blocked the effects of rhMIF on EMT and VMs formation. Collectively, our results support a critical role for the MIF-CXCR4 signaling axis in regulating hypoxia-induced VMs formation, indicating the potential usefulness of MIF as a notable target for the anti-vascularization treatment of GBM.

## INTRODUCTION

Glioblastoma multiforme (GBM) is the most common and deadly type of malignant brain tumor. Although individually variable, the average overall survival for patients with glioblastoma has improved slowly in the standard of care [1]. A prominent feature of GBM is severe hypoxia and vascular proliferation [2]. Targeting angiogenesis, especially by anti-VEGF agents, was considered a hopeful strategy to overcome the malignant progression of GBM. However, anti-angiogenic therapies were well tolerated in patients with GBM and clinical trial evidence indicated that it had no significant effect on prolonging long-term survival for patients with primary GBM [3]. The mechanisms of resistance to anti-angiogenic therapies are complicated and poorly understood. There must be some salvage-neovascularization instead of angiogenesis to rescue and contribute to the malignant progression of GBM under hypoxic conditions. Therefore, a better understanding of the molecular mechanisms of salvage-neovascularization is needed to develop more specific therapeutic strategies.

Increasing research has indicated that hypoxia is a well-established inducer of neovascularization [4, 5]. In addition to angiogenesis, the predominant process of neovascularization, vasculogenic mimicry (VM), de novo vascular networks formed by malignant cancer cells themselves, is also recognized [6]. Since the initial conceptualization of tumor cell VM, it was identified in various malignant tumors, including GBM [7–10]. Several studies revealed that VM is essential for the malignant behavior of gliomas to provide increasing amounts of blood flow for nutrient and oxygen supply [11]. Tumor cells that are capable of VM share a plastic phenotype that could be regulated by hypoxia [12]. Consistently, recent research has demonstrated that the hypoxia-promoted epithelial-to-mesenchymal transition (EMT) of GBM is strongly associated with the formation of VM [13, 14]. These findings suggested that VM could play a key role in adaption to the hypoxic environment in GBM. However, the detailed mechanism underlying the relationship between hypoxia and VM in glioma cells remains poorly understood.

Macrophage migration inhibitory factor (MIF) is a structurally unique cytokine with pro-inflammatory and tumor-promoting functions [15]. Specifically, MIF is highly expressed in various types of tumors, including prostate [16], colon [17], melanoma [18] and glioblastoma [19, 20]. In recent years, the significant role of MIF in tumorigenesis has been revealed; MIF expression is correlated with the stage and metastatic spread of GBM [21, 22]. Intriguingly, under hypoxia, the expression and secretion of MIF were strongly increased and promoted angiogenesis in GBM [23]. Notably, previous studies have shown that CXCR4, as a potential receptor of MIF, was a component of the axis inducing EMT in GBM [24]. Furthermore, CXCR4 is regulated by hypoxia-inducible factor 1 (HIF1) and implicated in invasion, metastasis and angiogenesis in a wide range of malignant tumors [25]. Those findings led us to hypothesize that overexpression of MIF and CXCR4 synergistically contributes to adaptation and progression within hypoxic tumor regions in GBM. However, whether MIF-CXCR4 is involved in hypoxia-induced VM formation by GBM cells remains to be explored.

In this study, we highlighted the function of MIF in the process of hypoxia-induced VM formation in GBM cells. Immunochemical studies of specimens of glioma patients revealed that hypoxia-induced overexpression of MIF and CXCR4 co-localized with VM. In addition, we confirmed that the MIF-CXCR4-AKT pathway was involved in the underlying molecular mechanism. Our results described a MIF-CXCR4 signaling axis regulating hypoxia-induced VMs formation to adapt to the tumor hypoxia microenvironment in GBM.

## RESULTS

### The expression of MIF and CXCR4 is correlated with HIF1α level in glioma specimens

**Figure 1:**
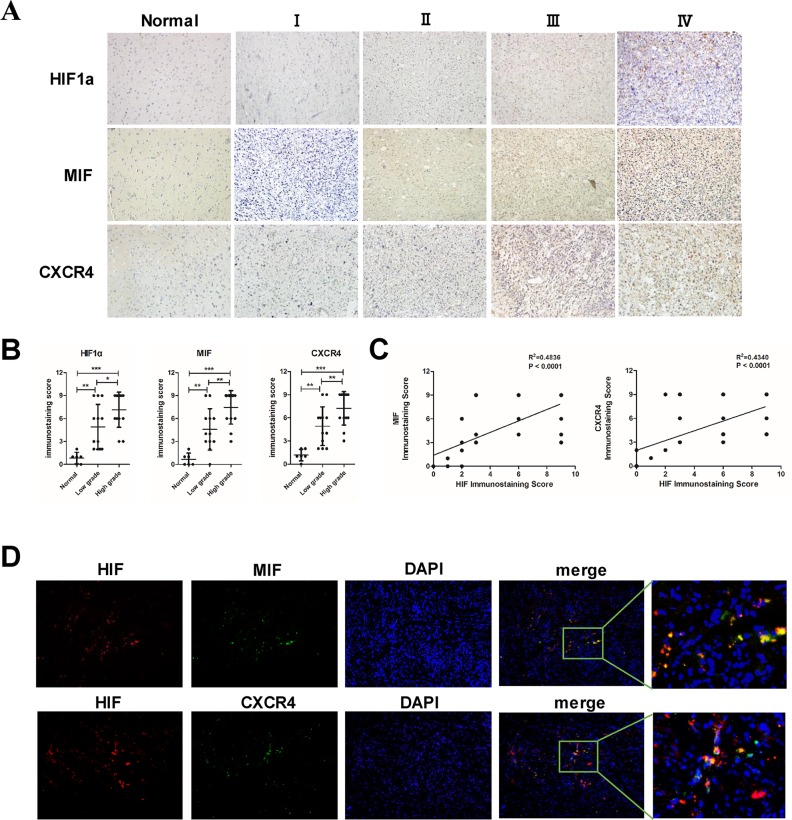
The expression of MIF and CXCR4 is positively correlated with HIF1α in glioma tissues **(A)** Representative images of normal brain tissues and different grades of glioma tissues showed the immunostaining of HIF1α, MIF and CXCR4 (magnification, 200×). **(B)** Statistic data of the immunostaining scores were analyzed showing the correlation of the expression of proteins with the grade of gliomas. **(C)** Correlation of HIF1α expression with MIF and CXCR4 was analyzed in 25 glioma specimens. The linear regression coefficient and statistical significance was indicated the positive correlation of HIF1α with MIF and CXCR4. **(D)** Double immunofluorescence staining were performed and merged to show co-localization (magnification: 200×). A large view of the co-localization was shown. HIF1α was labeled with red fluorescence. MIF and CXCR4 were labeled with green fluorescence. *P < 0.05, **P < 0.01, ***P< 0.0001, one-way ANOVA or linear regression.

We used immunohistochemistry to evaluate the expression of HIF1α, MIF and CXCR4 in our glioma specimens. The expression of HIF1α, MIF and CXCR4 in clinical specimens (six normal brain tissues and twenty-five human glioma tissues) was positively correlated with the grade of glioma (Figure 1A, 1B). Further linear regression analysis showed that the expression of MIF and CXCR4 were positively correlated with the level of HIF1α individually (Figure 1C). To illustrate that hypoxia is a direct inducer of MIF and CXCR4, we performed double immunofluorescence staining in GBM specimens to detect the co-localization of HIF1α with MIF and CXCR4. MIF and CXCR4 were highly expressed and co-localized within the hypoxic region indicated by HIF1α (Figure 1D). These data demonstrated that MIF and CXCR4 could be highly co-expressed within hypoxic regions of human gliomas.

### VM formation is correlated with the high co-expression of MIF and CXCR4 in glioma tissues

**Figure 2:**
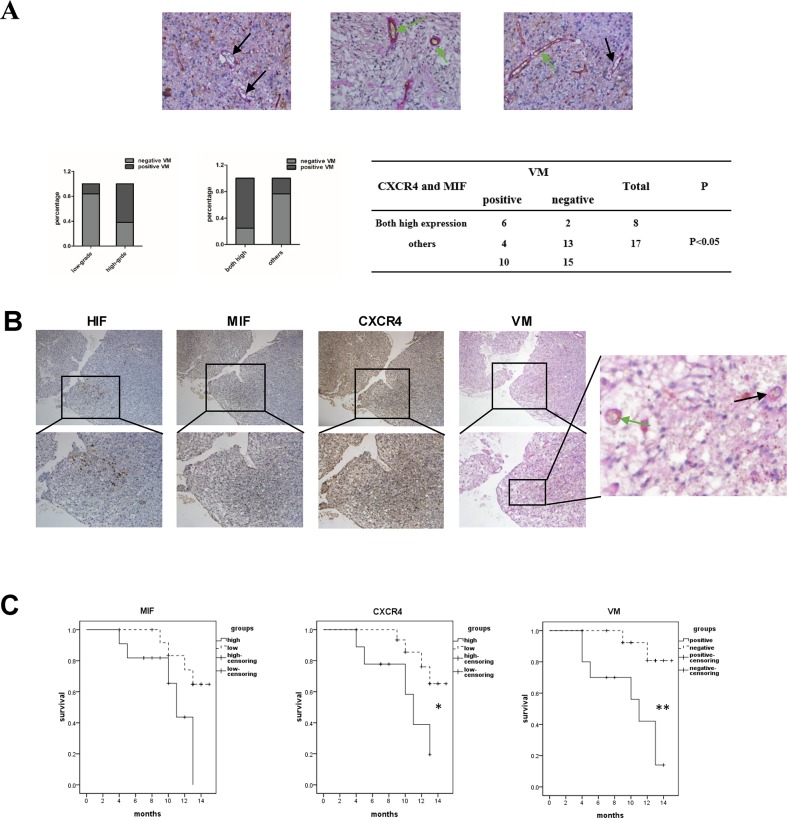
VM formation is correlated with the high co-expression of MIF and CXCR4 in glioma tissues **(A)** Representative images showed the VMs (black arrow) and vessels (green arrow) in glioma speicimens. There were red blood cells in the center of the VMs and vessels (magnification: 400×). Basement membrane-like structures were stained by PAS (pink) and endothelial cells were stained by CD34 (brown). Correlation of glioma grades and VM, MIF and CXCR4 high co-expressed glioma specimens and VM were analyzed by Fisher's exact test. **(B)** Immunohistochemistry was performed to detect HIF1α, MIF and CXCR4 in serial sections of GBM (the top row 100X, the bottom row 200X, a large view of the tumor section was to indicate the VM). **(C)** Kaplan–Meier analysis of survival for 25 glioma patients stratified by MIF, CXCR4 expression or VM-positive. Low = patients with immunostaining score less than 9. High = patients with immunostaining score equal to 9. A Wilcoxon rank sum test was used to compare survival curves. *P < 0.05; **P < 0.01.

After verifying the correlation of HIF1α and MIF with CXCR4 in gliomas, we further explored whether hypoxia-induced high co-expression of MIF and CXCR4 was correlated with the formation of VMs. We identified the VMs and endothelium-dependent vessels in the gliomas by double staining for PAS and CD34 (Figure 2A). At the same time, VM-positive specimens were quantified and analyzed. The results demonstrated that high-grade gliomas had more VM-positive specimens than did low-grade gliomas and that VM was closely associated with high co-expression of MIF and CXCR4 in gliomas. Further, we detected the spatial correlation of HIF1α, MIF, CXCR4 and VM in serial sections from GBM specimens (Figure 2B). There was significant co-localization of those four factors, suggesting that hypoxia-induced high co-expression of MIF and CXCR4 may play an important role in the formation of VM.

Finally, to evaluate the clinical significance of MIF, CXCR4 and VMs in glioma, overall survival was analyzed by stratifying patients according to the immunochemistry score for MIF and CXCR4 or VM-positive (Figure 2C). In our study, MIF levels were not significantly associated with survival. In contrast, the high expression of CXCR4 was associated with decreased patient survival. The most significant difference in survival was observed in VM-positive patients, who had more events of high co-expression of MIF and CXCR4 than did the VM-negative patients. Therefore, we proposed that the MIF-CXCR4 pathway potentially participated in the process of VM formation within hypoxic regions of gliomas to support malignant progression.

### Hypoxia increases MIF and CXCR4 expression and induces EMT and VM formation in GBM cells

**Figure 3:**
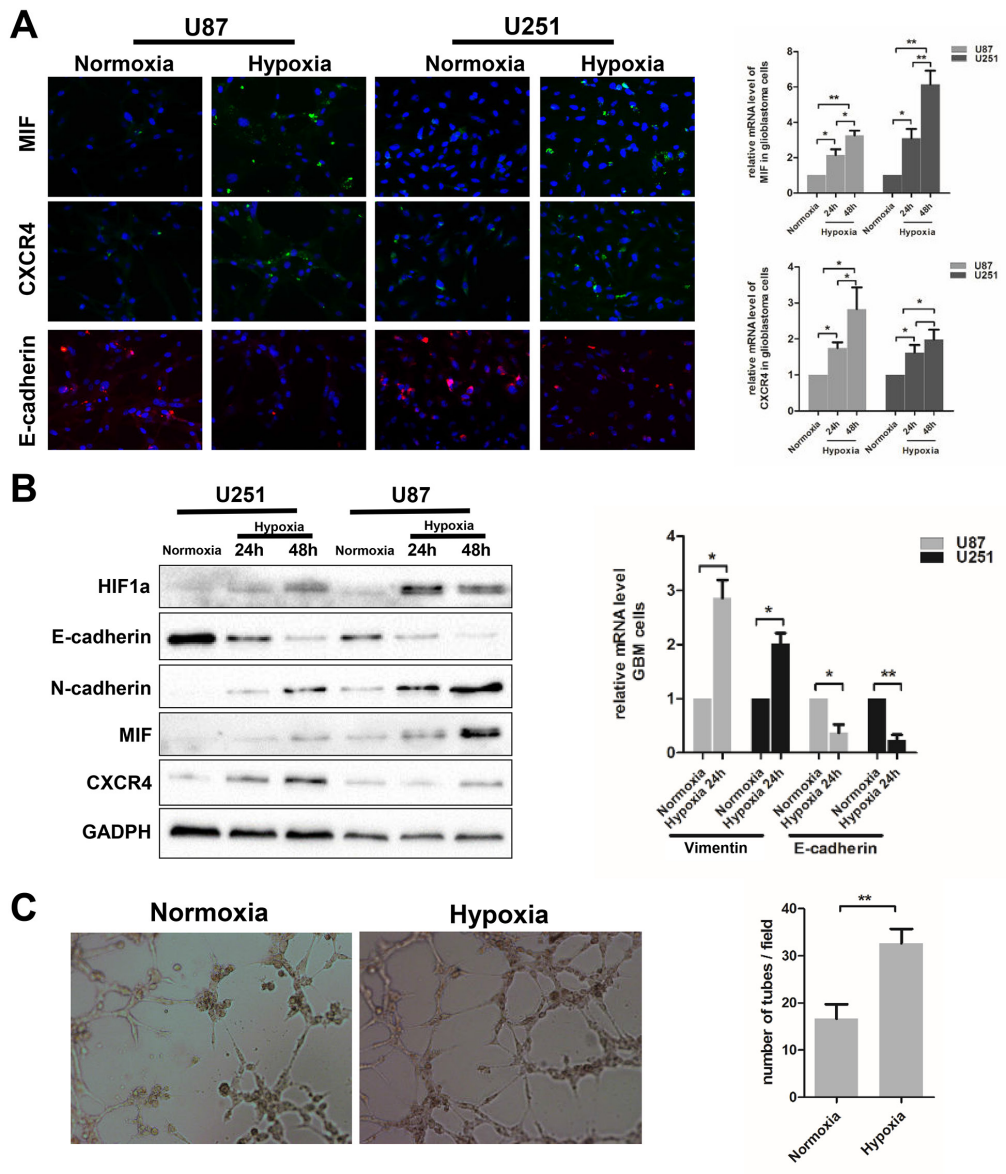
Hypoxia induces EMT and VM formation in glioblastoma cells **(A)** Immunofluorescence was analyzed for MIF, CXCR4 and endothelial marker (E-cadherin) in U87and U251cells exposed to hypoxia. Images obtained at magnification 200X. Relative mRNA expression levels of MIF and CXCR4 were detected by Q-PCR. **(B)** Western blots showed the expression levels of MIF, CXCR4 and multiple markers (E-cadherin, N-cadherin, Vimentin) associated with EMT in U87 and U251 cells under hypoxic conditions. Relative mRNA expression levels of E-cadherin and Vimentin were detected by Q-PCR. The data shown are the mean ± SD of independent experiments, n = 3. **(C)** VM tube formation assay was performed 6h with U87 cells under hypoxia and normoxia. Images were shown at magnification 400X. Numbers of tubes per file are shown. The data shown are the mean ± SD of independent experiments, n = 3. *P < 0.05; **P < 0.01 Student's 2-tailed t test or one-way ANOVA.

To further clarify that hypoxia regulates EMT and VM formation in GBM, experiments were designed in GBM cells. The expression of MIF and CXCR4 was examined by immunofluorescence and q-PCR in both U87 and U251 cells under hypoxic and normoxic conditions. Hypoxia significantly increased MIF and CXCR4 expression in U87 and U251 cells at both the protein and mRNA levels (Figure 3A). Further, western blot analysis was performed to show the expression of MIF, CXCR4 and several EMT markers in U87 and U251 cells under hypoxia compared to normoxia (Figure 3B). The expression of HIF1α, MIF and CXCR4 was strongly induced by hypoxia together with the mesenchymal markers N-cadherin and Vimentin. In contrast, the epithelial marker E-cadherin was dramatically decreased and little or no expression was detected in hypoxic conditions (Figure 3A, 3B). At the same time, compared with the normoxic conditions, we detected by q-PCR a higher expression of N-cadherin mRNA and a lower expression of E-cadherin mRNA (Figure 3B). Furthermore, to confirm the effect of hypoxia on the formation of VM, the VM tube formation assay was used to test the ability of VM formation by GBM cells *in vitro*. While U87 cells retained the ability to form VMs under normoxia, we found that U87 cells formed more stable VMs under hypoxia (Figure 3C). However, in U251 cells, no induction in VM formation was observed, even under hypoxia (data not shown). To further confirm the effect of hypoixa on VM formation of glioma cells, we used A172, one of glioma cell lines, to repeat the VM formation assay. The results showed that hypoxia significantly increased the number of VMs formation of A172 cells (Supplementary Figure 1A). Based on this result, further research mainly focused on U87 cells. These results demonstrate that hypoxia plays a role in enhancing the EMT and promoting VM formation by GBM cells.

### MIF influences the EMT and VM formation in GBM cells

**Figure 4:**
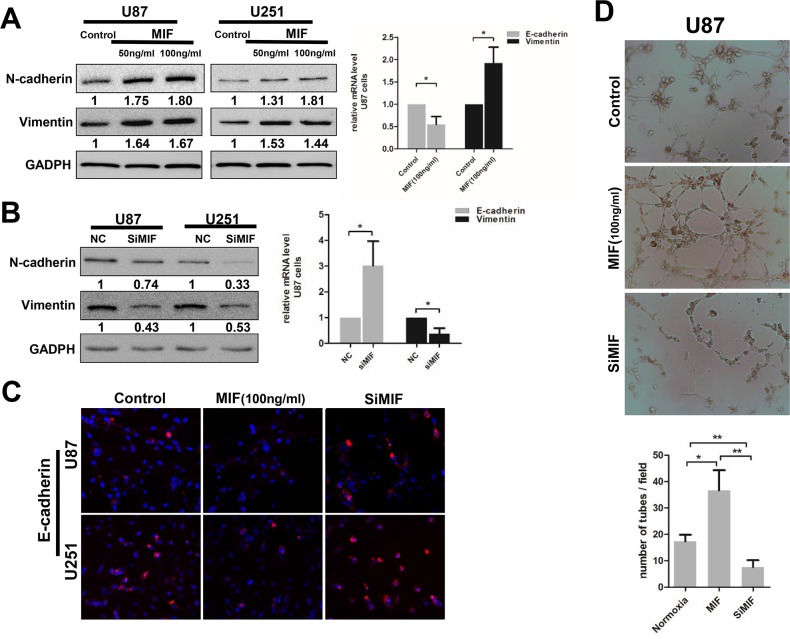
MIF enhances the EMT and VM formation in GBM cells **(A/B)** Western Blots were performed to analyze the expression level of mesenchymal markers (N-cadherin, Vimentin) in U87 and U251 cells treated with ectogenic rhMIF (50ng/ml, 100ng/ml) for 24h or transfected with siMIF. Quantitative relative analyses were calculated following Imagej analysis. The mRNA levels of EMT markers were also detected by Q-PCR. The data shown are the mean ± SD of independent experiments, n = 3. **(C)** Representative images of immunofluorescence showed the expression levels of E-cadherin in U87and U251cells treated with ectogenic rhMIF (100ng/ml) and transfected with siMIF compared to control (magnification 200X). **(D)** VM tube formation assay was performed with U87 cells as indicated. Representative images were shown at magnification 400X. Numbers of tubes per file are shown. The data shown are the mean ± SD of independent experiments, n = 3. NC, negative control. *P < 0.05, **P < 0.01, Student's 2-tailed t test or one-way ANOVA.

In glioma specimens, we found that VM formation was closely associated with high co-expression of MIF and CXCR4. Because CXCR4 is a cell surface chemokine receptor, we explored whether MIF could promote VM formation by individual GBM cells. To verify the effect of MIF on EMT and VM formation in GBM cells, ectogenic rhMIF and siMIF were carried out to determine the influence of MIF overexpression and intrinsic MIF knock-down on VM formation by GBM cells. The expression of mesenchymal markers (N-cadherin and Vimentin) and epithelial markers (E-cadherin) was used to detect the EMT of GBM cells. Exposure to rhMIF resulted in a significant increase in N-cadherin and Vimentin expression mRNA and protein in a concentration-dependent manner (Figure 4A), suggesting that rhMIF could induce the EMT of GBM cells. Consistent with the effect of EMT, rhMIf increased the migration of GBM cells (Supplementary Figure 2A, 2B, 2C). The siMIF transfection efficiency was evaluated by quantitative real-time PCR (q-PCR) and western blotting (Supplementary Figure 1B). Conversely, there were lower N-cadherin and Vimentin mRNA and protein levels in transfected cells compared to the control (Figure 4B). Consistently, the epithelial marker E-cadherin showed lower expression in rhMIF stimulated cells and higher expression in siMIF transfected cells by immunofluorescence (Figure 4C). Moreover, U87 cells transfected with siMIF had a less elongated morphology than did the negative control cells, suggesting a repression of the EMT (Supplementary Figure 2A). Finally, we repeated the VM tube formation assay to confirm the role of MIF on VM formation. More VMs of U87 cells were established under the rhMIF stimulation compared to the control group, while the capacity of VM formation was lower in siMIF transfected U87 cells (Figure 4D). All of the data demonstrated that MIF positively regulates EMT and VM formation in GBM cells. These data suggest that MIF indeed affected the capability of VM formation in GBM cell lines.

### Hypoxia regulates VM formation through the MIF-CXCR4-AKT-EMT pathway in GBM cells

**Figure 5:**
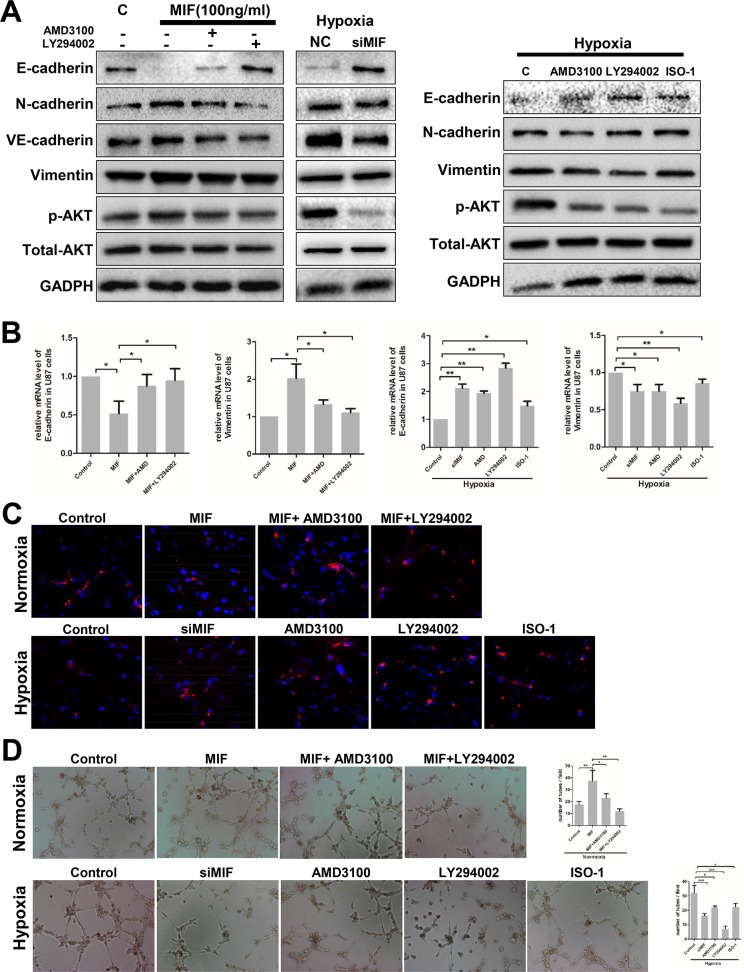
Hypoxia induces VM formation through the MIF-CXCR4-AKT-EMT pathway in glioblastoma cells **(A)** EMT markers, P-AKT and total AKT expression levels were detected by western blot in U87 cells treated with rhMIF and AMD3100 (a CXCR4 antagonist, 200 nM) or LY294002 (a PI3K inhibitor, 10 μg/ml). Western blots were also used to analyze the expression levels of proteins in U87 cells transfected with siMIF and treated with AMD3100, LY294002, or ISO-1(a MIF antagonist, 20 ug/ml) under hypoxia. **(B/C)** Q-PCR and immunofluorescence were used to detect the mRNA and protein levels of E-cadherin in U87 cells with different treatments as indicated. The data shown are the mean ± SD of independent experiments, n = 3. **(D)** U87 cells with the same treatments as (c/d) were analyzed by the VM tube formation assay. Representative images are shown at 400X magnification. Numbers of tubes per file are shown. The data shown are the mean ± SD of independent experiments, n = 3. NC, negative control; C, control. *P < 0.05, **P<0.01, one-way ANOVA.

Initially, MIF stimulation was performed in a time-dependent and concentration-dependent manner. We determined that AKT was activated by ectogenic rhMIF in a time-dependent but concentration-independent fashion in U87 cells. U87 cells transfected with siMIF had an impaired phosphorylation level of AKT compared to the control (Supplementary Figure 3A). To determine whether the CXCR4-AKT-EMT pathway was underlying MIF-induced VMs, experiments were designed to discover whether MIF-induced EMT could be impeded by inhibiting the pathway. Western blots, q-PCR and immunofluorescence were used to analyze the EMT marker expression and the activity of the AKT pathway. In normoxia, AMD3100 (a CXCR4 antagonist) or LY294002 (a PI3K inhibitor) together with MIF were added to the regular culture media of U87 cells. AMD3100 and LY294002 significantly impaired the effect of MIF on EMT and the phosphorylation of AKT, which demonstrated that MIF regulated EMT through CXCR4-AKT (Figure 5A, 5B, 5C). In other experiments, under hypoxia, U87 cells were transfected with siMIF and treated with AMD3100, LY294002, or ISO-1 (an MIF antagonist). Knock-down of endogenic MIF by siMIF, neutralizing ectogenic MIF by ISO-1 or blocking the CXCR4-AKT pathway disrupted the hypoxia-induced EMT, showing that the MIF-CXCR4 pathway plays a key role in hypoxia-induced EMT in GBM cells (Figure 5A, 5B, 5C). We observed the same results in the U251 cell line by immunofluorescence (Supplementary Figure 3B).

Finally, VM tube formation assays were executed utilizing the same treatments as above. In normoxia, AMD3100 and LY294002 significantly impaired MIF-induced VMs formation. More importantly, perturbations at each step of the MIF-CXCR4-AKT pathway obviously suppressed the hypoxia-induced VMs formation in U87 cells (Figure 5D). Thus, it appears that MIF regulates hypoxia-induced VM formation through the CXCR4-AKT-EMT pathway in GBM cells.

### MIF promotes the EMT in GBM cells and tumor growth *in vivo*

**Figure 6:**
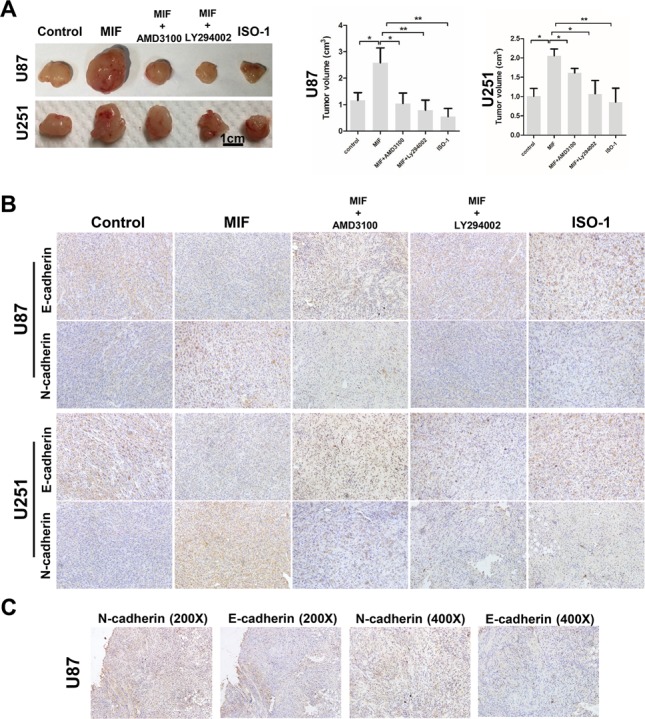
MIF promotes the EMT and VM formation by GBM cells *in vivo* **(A)** Two arrays of representative U87 and U251 cell xenografts were shown. U87 and U251 cells were injected into mice subcutaneously. After the mice were killed, U87 and u251 cell xenografts were measured by tumor volume. The data shown are the mean ± SD, n = 3. **(B)** Immunohistochemistry reveals the expression of N-cadherin and E-cadherin in different groups of xenografts (magnification 200X). **(C)** Two serial sections of the MIF group of U87 cell xenografts were used to confirm the expression of N-cadherin and E-cadherin. *P < 0.05, **P<0.01, one-way ANOVA.

To extend our findings *in vivo*, we used both U87 and U251 cells to establish glioma xenograft models in mice and selected 15 tumors of almost the same size to randomly divide into groups. To further test whether the CXCR4-AKT pathway was involved in the MIF-induced EMT, five groups (control, MIF, MIF and AMD3100, MIF and LY294002, and ISO) were established. Then, subcutaneous injections were carried out twice a week for three weeks. As expected, there were significantly higher volumes of subcutaneous tumors in the MIF group compared to the other groups (Figure 6A), indicating that *in vivo*, MIF contributes to tumor progression and could be inhibited by blocking CXCR4-AKT pathway activity. After the mice were euthanized, the expression of N-cadherin and E-cadherin was determined by immunohistochemical studies of tumor sections from each group (Figure 6B). We found the same effect of MIF in xenograft tumors as for the *in vitro* cell experiments; that is, MIF induced the EMT in GBM. In addition, two serial sections of U87 tumors in the MIF groups were stained with the EMT marker to confirm the transfer of EMT (Figure 6C). Taken together, our data provide further evidence for a link between the MIF and CXCR4-AKT pathway in mediating the EMT in GBM.

## DISCUSSION

Hypoxia has been recognized as a major feature of GBM [26]. In response to hypoxia, GBM highly expresses pro-angiogenic cytokines and induces microvascular proliferation [27]. Highlighting the potential value of a therapeutic strategy targeting angiogenesis, many VEGF targeting agents have been explored in GBM [28]. However, the overall survival of the patients was not significantly improved. To date, many studies have revealed that VM is a brand-new tumor vascular supplement independent of angiogenesis [29]. VMs could be a critical element for resistance to anti-VEGF targeting therapy. In this study, we established a previously unrecognized link between VM formation and high co-expression of MIF and CXCR4 within hypoxic regions of glioma specimens. Additional data revealed that MIF promotes hypoxia-induced VM formation individually through the CXCR4-AKT-EMT pathway. Our results implicated the crucial role of MIF and its downstream pathway in providing an additional blood supply for aggressive tumor growth and adaption to a severe environment.

Many studies indicated that VM formation was related to hypoxia-related signaling pathways and metastatic phenotype [30–33]. The hypoxia-induced over-expression of MIF and CXCR4 has been separately reported to mediate the invasion of GBM [34, 35]. Here, we determined that the expression of MIF and CXCR4 was positively correlated with the grade of glioma and the HIF level. Furthermore, a closely positive relationship between VM formation and high co-expression of MIF and CXCR4 was revealed within hypoxic regions of gliomas. Although the MIF has been reported as an independent predictive factor for the prognosis of glioma patients [35], our study showed no significant difference in overall survival according to the expression of MIF. This different result may be caused by the limited number of clinical specimens. Of note, VM was a significantly unfavorable prognostic factor among glioma patients. Intriguingly, most VM-positive patients have high co-expression of MIF and CXCR4. Those clinical analyses provided evidence to support our hypothesis that the overexpression of MIF and CXCR4 synergistically promotes hypoxia-induced VM formation.

The normal developmental process of EMT contributes to tumor cell plasticity, by which epithelial cells transform into mesenchymal cells. It has been reported to exist in various tumors as a characteristic of the metastatic phenotype. Interestingly, EMT was implicated in VM formation of various malignant tumors [36–39]. Consistently, we found that GBM cells were induced in EMT by hypoxia and formed more VMs. These findings indicated that EMT participated in hypoxia-induced VM formation in GBM [13]. However, although the EMT response of U251 cells to hypoxia seemed the same as in U87 cells, the U251 cells failed to form VM *in vitro*. This may result from the characteristics of the cells themselves. U87 cells have a more mesenchymal phenotype even in normoxic conditions and were recognized as a good model to investigate VM formation in previous studies [40].

Overexpression of MIF consistent with the features of EMT in pancreatic cancer cell lines was reported by Naotake Funamizu et al. [41]. In our study, we found that the EMT and VM formation by GBM cells was induced directly by ectogenic rhMIF in normoxia and inhibited by transfected siMIF both in normoxia and hypoxia. Those findings demonstrated that MIF serves as a mediator of hypoxia-induced VMs formation via EMT. Owing to the link between VM formation and high co-expression of MIF and CXCR4 in specimens, AMD3100 was used to antagonize the function of CXCR4. The effect of MIF on EMT and VM formation was reversed by AMD3100. Combined with our clinical data, we are convinced that CXCR4 is downstream of MIF in promoting hypoxia-induced VM formation. Some studies demonstrated that CXCR4 is a receptor of MIF [42, 43]; however, whether MIF directly binds to CXCR4 in GBM requires further research.

Activated CXCR4 was described by Gai to activate the phosphoinositol-3-kinase (PI3K) and mitogen activated protein kinase (MAPK) signaling pathways [44, 45]. Recent studies showed that CXCR4 play an important role in promoting EMT process of tumor cells, including GBM [46–48]. And phosphorylation of ERK1/2, AKT and increasing expression of EMT-related transcription factors were involved in regulation of CXCR4-induced EMT in tumors, including GBM [24, 49–52]. Here, through intervention with different antagonists, we confirmed that hypoxia-induced VM formation was dependent on a MIF-CXCR4-AKT-EMT pathway. Moreover, through analyzing TCGA data, we found the expression of MIF was possitively correlated with SNAI1 which was a transcription factor that promoting EMT process (Supplementary Figure 3C). Additionally, *in vivo*, the xenografts stimulated with ectogenic rhMIF become more progressive, consistent with previous research [53]. More importantly, the MIF group expressed higher levels of mesenchymal markers. These data revealed that MIF acts as a key mediator of hypoxia-induced VM formation via the CXCR4-AKT-EMT pathway. However, whether there is another mechanism linking MIF and VM in addition to EMT remains to be further investigated.

**Figure 7:**
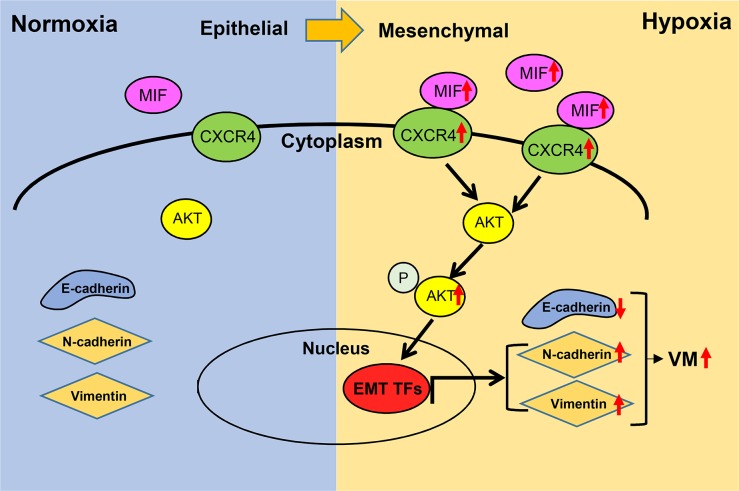
Schematic diagram of MIF promoting hypoxia-induced VM formation through CXCR4/AKT/EMT pathway Hypoixa increases the expression of MIF and CXCR4 respectively leading to the activation of CXCR4 and the phosphorylation of AKT. The activation of AKT induce overexpression of EMT associated trascription factors which could increase the expression of mesenchymal markes. Finally, the EMT could increasing the ability of glioma cells in VM formation.

In conclusion, we proved that MIF plays a crucial role in hypoxia-induced VM formation in GBM (Figure 7). Moreover, we recognized a novel link between the expression of MIF and CXCR4 and VM formation in GBM. Our study further demonstrated that hypoxia co-upregulates MIF and CXCR4 and activates the MIF-CXCR4-AKT-EMT axis to induce VM formation. Hence, MIF is a well-defined therapeutic target contributing to GBM malignant progress. Notably, its downstream pathway might provide a possible strategy for improving the prognosis for GBM patients.

## METHODS

### Ethics statement

The study has been conducted in accordance with the ethical standards and according to the Declaration of Helsinki and national and international guidelines. This study was approved by the Institutional Review Board of Shandong University. Written informed consent was obtained from all patients, and the hospital ethics committee approved the experiments.

### Tissues samples and cell lines

Six normal brain tissues were collected from patients undergoing internal decompression surgery following severe traumatic brain injury. Twenty-five human glioma tissues, including twelve low-grade glioma tissues (five grade-I tumors and seven grade-II tumors) and thirteen high-grade glioma tissues (three grade-III tumors and ten grade-IV tumors) were obtained from the Department of Neurosurgery, Qilu Hospital of Shandong University. Glioma specimens were verified and classified by two experienced clinical pathologists according to the WHO standard classification of tumors. Written informed consent was obtained from all patients, and the hospital ethics committee approved the experiments.

Human glioma cell lines (U87and U251) were purchased from the Chinese Academy of Sciences Cell Bank (Shanghai, China). The cell lines were grown in Dulbecco's modified Eagle's medium (DMEM, SH30022.01B, Hyclone, UT, USA) supplemented with 10% fetal bovine serum (10082147, Gibco, MD, USA) in a humidified incubator with 5% CO_2_ at 37°C. For hypoxia treatment, cells were first maintained in the regular normoxic incubator for around 16 h until the cells attached to the flasks. Following this the flasks were transferred to an incubator chamber flushed with a gas mixture containing 1% O_2_, 5% CO_2_ at 37°C.

### Chemical reagents, siRNA and transfections

rhMIF [Peprotech (300-69), NJ, USA], AMD3100 [Selleck chemicals (S8030), TX, USA], LY294002 [Beyotime (S1737), Shanghai, China], ISO-1[Biovision (1895-5), CA, USA] and DAPI [Beyotime (C1005), Shanghai, China] were obtained from indicated companies. SiRNA negative control and siMIF were designed and purchased from Gene Pharma (Shanghai, China). The siRNA sequences are listed in Online Resource 3. Cell transfection and co-transfection experiments were performed with nucleic acids using Lipofectamine 2000 [Life Technologies (11668-019), CA, USA] according to the manufacturer's instructions.

### Immunohistochemistry

Paraffin-embedded consecutive tissue sections were sliced and mounted on microscopic slides. Heat-induced epitope retrieval was performed with a microwave in 10 mmol/L citric acid buffer at pH 7.2. The samples were incubated with the antibody overnight in a humidified chamber at 4°C followed by incubation with a horseradish peroxidase-conjugated secondary antibody [ZSGB-BIO (PV-9000), Beijing, China]. Primary antibodies used: Rabbit anti-MIF [1:200 dilutions, Proteintech (20415-1-AP), Wuhan, China], anti-CXCR4 [1:200 dilutions, Abcam (ab1670), Cambridge, UK] and anti-HIF1α [1:200 dilutions, Proteintech (20960-1-AP), Wuhan, China] antibodies. Finally, 3, 3′-diaminobenzidine tetrahydrochloride (DAB) was used to reveal the signal. The total immunostaining score was estimated using both the percentage of positively stained tumor cells and the staining intensity. The percentage positivity was scored as “0” (<5%, negative), “1” (5–25%, sporadic), “2” (25–50%, focal), or “3” (>50%, diffuse). The staining intensity was scored as “0” (no staining), “1” (weakly stained), “2” (moderately stained), or “3” (strongly stained). Both the percentage of positive cells and the staining intensity were evaluated under double-blind conditions. The immunostaining score was calculated as the percentage positive score multiplied by the staining intensity score and ranged from 0 to 9. Based on the immunostaining score, the glioma patients were divided into two groups: the low expression group (0–6) and the high expression group (9).

### Immunofluorescence

Cells cultured on 24-well plate were washed with phosphate-buffered saline (PBS) and fixed in 4% paraformaldehyde for 10 min. After 3 times washing with PBS, cells were permeabilized with 0.2% Triton [Beyotime (ST795), Shanghai, China] in PBS, washed again with PBS followed by a blocking step for 30 min with normal goat serum. Cells were incubated overnight at 4°C in primary antibodies. Primary antibodies used: Mouse anti-HIF1α [1:200 dilutions, Abcam (ab1), Cambridge, UK] and Rabitt anti-CXCR4 [1:200 dilutions, Abcam (ab1670), Cambridge, UK], anti-MIF [1:100 dilutions, Santa Cruz (sc-20121), TX, USA], anti-E-cadherin [1:100 dilutions, Proteintech (20648-1-AP), Wuhan, China]. On the following day, glioma cells were washed in PBS 3 times and then incubated in the dark for 1 h with the appropriate secondary antibodies: goat anti-Rabbit Alexa 488 (ZSGB-BIO (ZF-0511), Beijing, China), goat anti-Mouse Alexa 594 [ZSGB-BIO (ZF-0513)], goat anti-Rabbit Alexa 594 [ZSGB-BIO (ZF-0516)]. DAPI [Beyotime (C1005), Shanghai, China] staining was performed for 5 min.

Paraffin-embedded consecutive tissue sections were prepared as before with heat-induced epitope retrieval. Then, sections were washed 3 times for 5 min in PBS and blocked for 30 min in blocking solution (5% goat serum in PBS). Sections were then incubated overnight at 4°C in primary: both and Rabbit anti-CXCR4 or both Mouse anti-HIF1α and anti-MIF antibodies in PBS. After overnight incubation, sections were washed 3 times for 5 min in PBS and incubated with both anti-mouse and anti-rabbit secondary antibodies for 1h at RT in the dark. Sections were then washed 2 time for 5 min in PBS, DAPI was applied for 5 min, and followed by a final wash in PBS for 5 min. Images were acquired using an inverted Olympus microscope (DP72, Japan).

### Western blot

Total protein was extracted from tissues and cells using RIPA buffer [Beyotime (P0013B), Shanghai, China] with 1% phenylmethyl sulfonylfluoride, and Protein concentration was determined by the BCA method [Beyotime(23225)]. Proteins were separated using 10% SDS-PAGE and transferred onto polyvinylidene difluoride membranes [Millipore (ISEQ00010), MA, USA]. The membranes were blocked by 5% skim milk blocking buffer for 1 hour and then incubated in the primary antibodies at 4°C overnight. After washing with TBST, the blots were incubated with horseradish peroxidase-conjugated secondary antibodies at room temperature for 1 h. Finally, protein bands were visualized by enhanced chemiluminescence (ECL) [Millipore (WBKLS0100)] and detected using an ECL detection system (Thermo-Scientific, Beijing, China) and quantified with Imagej software. The following primary antibodies were used: Rabbit anti-E-Cadherin, N-Cadherin, Vimentin, VE-Cadherin, AKT, p-AKT (Ser473) were purchased from Cell Signaling Technology, MA, USA (3195, 13116, 5741, 2500, 4691, 9271). Mouse anti- CXCR4 was purchased from Santa Cruz Biotechnology, TX, USA (sc-53534). Rabbit anti- MIF antibody was purchased from Proteintech, Wuhan, China (20415-1-AP). Rabbit anti-GAPDH antibody was purchased from Goodhere Biotechnology Co. LTD, Hangzhou, China (AB-P-R 001).

### RNA isolation and quantitative real-time PCR

Total RNA was isolated using RNAiso Plus (9108, Takara). Total RNA (0.5–1 μg) was reverse-transcribed with a ReverTraAce qRT-PCR kit (FSQ-101, Toyobo) according to the manufacturer's protocol to synthesize cDNA. Real-time PCR was performed using a SYBR Premix Ex TaqTM Kit (QPK-201, Toyobo) with specific primers. The reactions were performed by using a Lightcycler 2.0 instrument (Roche Applied Science). The mRNA levels were normalized to GAPDH. All data for each sample were collected in triplicate. The fold changes were calculated by relative quantification (2^−ΔΔCt^). The specific primers used in the present study are listed in Supplementary Table 1.

### VM tube formation assay

VM tube formation was performed as described previously [40]. Briefly, 24-well tissue culture plate were coated with Matrigel Basement Membrane Matrix (0.1 ml/well, BD Bioscience, 356234) which was allowed to polymerase at 37°C for 1 h. Cells were resuspended and seeded on Matrigel at 2×10^5^ cells/ml, then incubated without serum in 5% CO_2_ at 37°C for 6 h. Cultures were photographed using an inverted Olympus microscope (DP72, Japan).

### Animal experiments

All animal experiments were performed in accordance with the NIH Guide for the Care and Use of Laboratory Animals. We performed all animal surgeries under ketamine anesthesia and took every effort to minimize animal suffering. Athymic nude mice (male; 4 weeks old; 20–30 g) were provided by Shanghai SLAC Laboratory Animal Co., Ltd (Shanghai, China). The mice were randomly divided into five groups. U87 cells (2 × 10^6^) and U251 cells (5 × 10^6^) in 100 μl of PBS were inoculated subcutaneously into the flanks of nude mice. When tumor volume reached 150–200 mm^3^, the mice were randomized and subcutaneously injected for 3 weeks with MIF (50 ug/kg), AMD3100 (200 uM/kg), LY294002 (10 mg/kg), ISO-1 (10 mg/kg) or PBS controls twice a week. Mice were sacrificed after 3 weeks.

### Statistical analyses

Data analyses were conducted with SPSS 16.0 (SPSS, IL, USA) and GraphPad-Prism5 (GraphPad, CA, USA). Data were analyzed using one-way ANOVA, Student's 2-tailed t test, linear regression analysis or Fisher's exact test. Data are presented as mean ± standard deviation (SD) of three independent experiments, followed by Dunnett's test for multiple comparisons of the means. All tests were 2-tailed, and p < 0.05 was considered statistically significant.

## SUPPLEMENTARY MATERIALS FIGURES AND TABLE


