# Supplementary material for: Macrophage migration inhibitory factor promotes vasculogenic mimicry formation induced by hypoxia via CXCR4/AKT/EMT pathway in human glioblastoma cells
Source: Oncotarget. 2017 Jun 27;8(46):80358–72. doi: 10.18632/oncotarget.18673 (PMC5655204; doi:10.18632/oncotarget.18673)
Supplement: Supplementary file 1 [file oncotarget-08-80358-s001.pdf]

Macrophage migration inhibitory factor promotes vasculogenic mimicry formation induced by hypoxia via CXCR4/AKT/EMT pathway in human glioblastoma cells

SUPPLEMENTARY MATERIALS

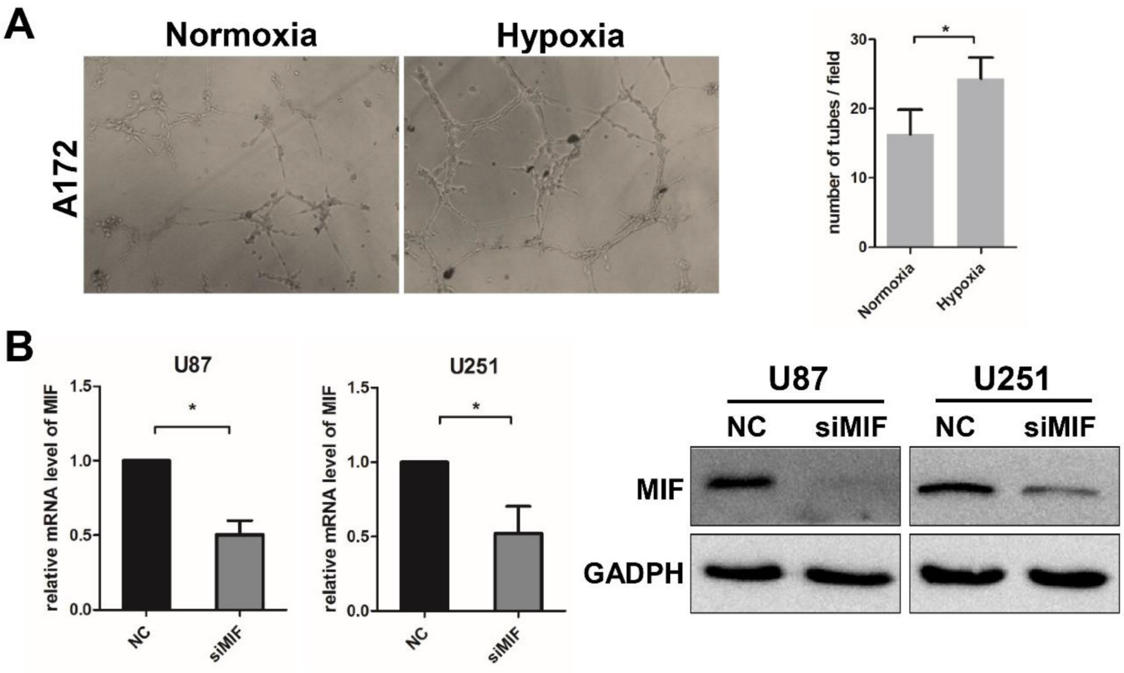

Supplementary Figure 1: Hypoxia promote VM formation of A172 and the transfection efficiency of siMIF. (A) VM tube formation assay was performed 6h with U172 cells under hypoxia and normoxia. Images were shown at magnification 400X. Numbers of tubes per file are shown. The data shown are the mean ± SD of independent experiments, n = 3. **(B)** U87 and U251 cells were transfected with 100 nM siMIF for 24 h. The cells were collected for q-PCR and western blot to quantify MIF expression. The data shown are the mean ± SD of independent experiments, n = 3. *P < 0.05 Student's 2-tailed t test.

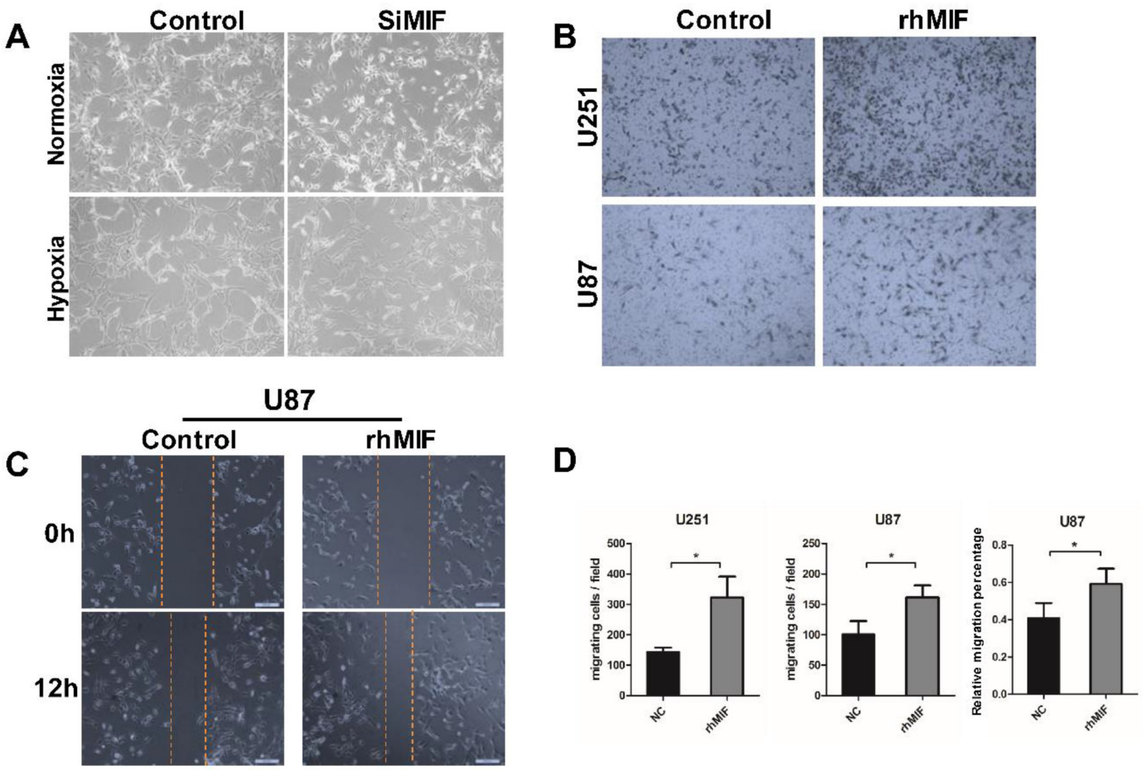

Supplementary Figure 2: Knock-down expression of MIF decreases the mesenchymal morphology of U87 cells and rhMIF increases the migration of GBM cells. **(A)** U87 cells were transfected with 100 nM siMIF for 24 h. Then, then images were captured using a microscope. (magnification 200X). **(B)** A total of $2X10^4$ U87 and U251 cells in FBS-free medium with 100ng/ml rhMIF were seeded into the upper chamber of an uncoated transwell. After 12 h, cells migrating on the lower surface were fixed and stained with crystal violet. Representative images from the quantification are shown (magnification 200X). **(C)** U87 cells were cultured in FBS-free medium with 100ng/ml rhMIF. Then, a wound was formed by scraping the cells with a 200μl tip. Images were captured at two time point (0 h, 12 h) by a microscopy (magnification 200X). The data shown are the mean ± SD of independent experiments, n=3. *P < 0.05, Student's 2-tailed t test.

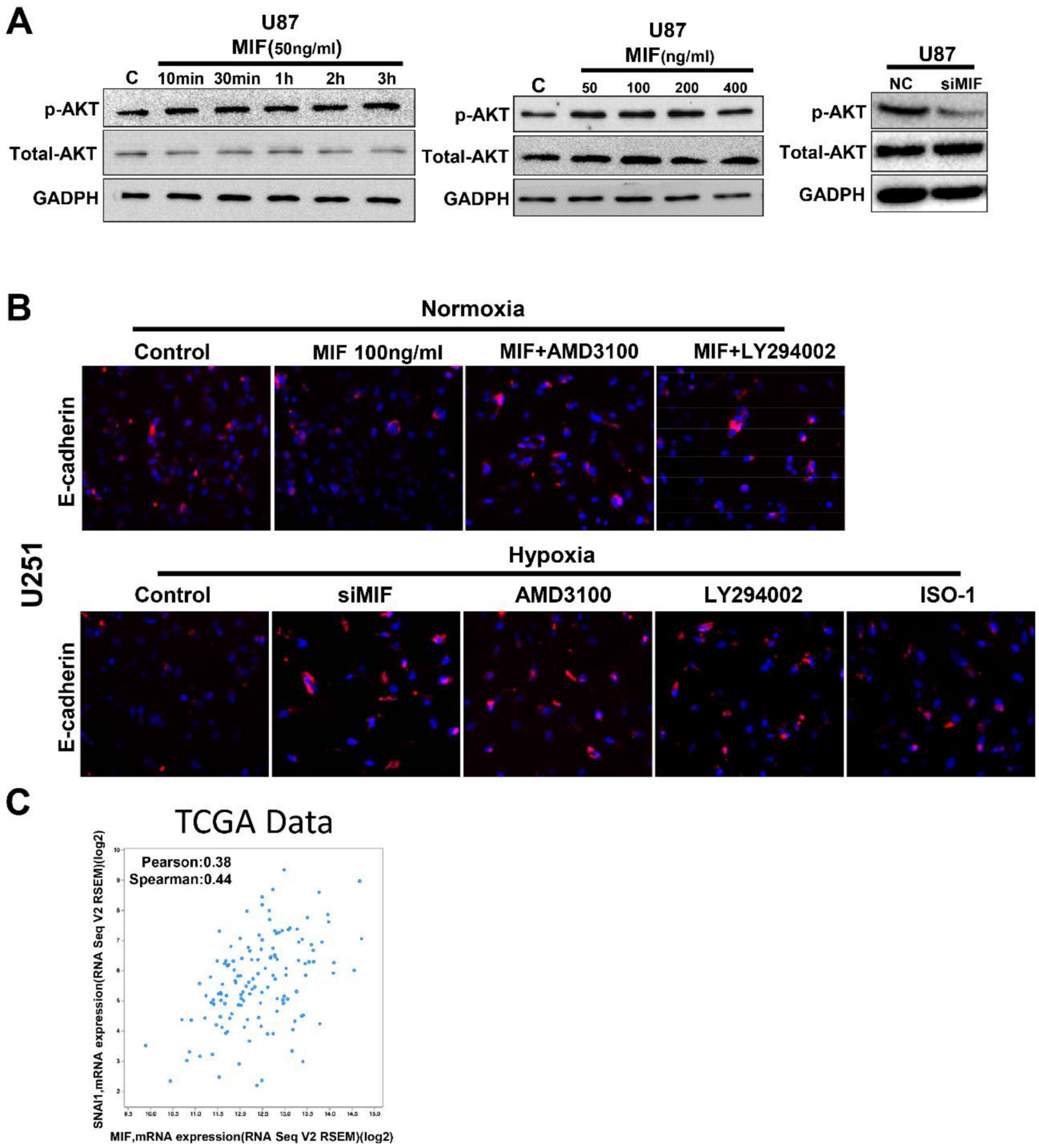

Supplementary Figure 3: The effect of rhMIF on AKT pathway of U87 and the E-Cadherin protein level in U251. **(A)** U87 cells were treated with ectogenic rhMIF in time and concentration gradients. P-AKT, total AKT and GAPDH levels were determined by western blot. U87 cells transfected with siMIF were also detected by western blot. **(B)** Immunofluorescence were performed to detect the mRNA and protein levels of E-cadherin in U87 cells with different treatment as indicated. **(C)** TCGA data was analyzed to detect the correlation of EMT related transcription factors and MIF. The results showed SNAI1 expression was positively correlated with MIF expression.

Supplementary Table 1: Primers and siRNA used for this study

Primers for q-PCR	Forward primer sequence(5'-3')	Reverse primer sequence (5'-3')
MIF	TGGAACAACTCCACCTTCGC	CCGTTTATTTCTCCCCACCAGA
CXCR4	ACGCCACCAACAGTCAGAG	AGTCGGGAATAGTCAGCAGGA
E-Cadherin	ATTTTTCCCTCGACACCCGAT	TCCCAGGCGTAGACCAAGA
Vimentin	AGTCCACTGAGTACCGGAGAC	CATTTCACGCATCTGGCGTTC

RNA oligo	Sequence(5'- 3')
Negative Control	**UUCUCCGAACGUGUCACGUTTACGUGACACGUUCGGAGAATT**
SiMIF	CCGAUGUUCAUCGUAAACATTUGUUUACGAUGAACAUCGGTT
